# Characterization of KLHL14 anti-oncogenic action in malignant mesothelioma

**DOI:** 10.1016/j.heliyon.2024.e27731

**Published:** 2024-03-09

**Authors:** Angelo Canciello, Reyes Benot Domínguez, Barbara Barboni, Antonio Giordano, Andrea Morrione

**Affiliations:** a Sbarro Institute for Cancer Research and Molecular Medicine and Center for Biotechnology, Department of Biology, College of Science and Technology, Temple University, Philadelphia, PA, 19122, USA; b Faculty of Bioscience and Technology for Food, Agriculture and Environment, University of Teramo, 64100, Teramo, Italy; c Department of Medical Biotechnologies, University of Siena, 53100, Siena, Italy

**Keywords:** KLHL14, Nuclear cytoplasmic shuttling, TGF-β, Tumor suppression, Malignant mesothelioma

## Abstract

Malignant mesothelioma (MM) is a very aggressive neoplasia with a short life expectancy and limited therapeutic options. Thus, the identification of novel molecular targets is a matter of great urgency. Kelch-like (KLHL) proteins play an important role in a number of physiological and pathological cell-regulatory processes. Among this family, the function of KLHL14 is still very poorly characterized. *KLHL14* was originally identified as a gene involved in regulating the epithelial-mesenchymal transition (EMT) process. Here, we demonstrate that KLHL14 not only prevents EMT but also plays an anti-oncogenic role in MM. Indeed, KLHL14 depletion enhanced proliferation, motility, invasion and colony formation in MM cells. Importantly, we also demonstrated that KLHL14 mechanism of action is dependent on Transforming Growth Factor β (TGF-β). In fact, TGF-β promotes *de novo* synthesis, increases protein stability and induces nuclear-cytoplasmic shuttling of KLHL14. Collectively, this research is an important step further to decipher KLHLs mechanism of action and further contributes to the understanding of the molecular mechanisms regulating MM.

## Highlights

• KLHL14 is expressed in mesothelioma subtypes-derived cells.
• KLHL14 localizes in nuclear and cytoplasmic compartments.
• TGF-β induces KLHL14 shuttling, increases *de novo* synthesis and KLHL14 stability.
• KLHL14 has anti-oncogenic action in mesothelioma cells.
• KLHL14 depletion promotes proliferation, migration, invasion and colony formation.

## 1 Introduction

Malignant Mesothelioma (MM) is an aggressive cancer originating mostly in the pleura (85% of cases, called malignant pleural mesothelioma, MPM), with a smaller fraction of about 10% originating in the peritoneum, and less than 5% developing in the pericardium or testis [1]. This rare tumor affects the layer of cells surrounding main organs, named mesothelium, and is histologically classified as epithelioid (50% of cases), sarcomatoid or fibrous (15%), and mixed or biphasic (30%) [2]. MM represents one of the deadliest malignancies, with a five-year survival rate of 10% [3,4]. This poor prognosis, combined with the late stage diagnosis and the lack of proven effective therapies make this cancer an important health challenge. Presently, exposure to asbestos fibers constitutes the main cause of MM, while zeolites, radiation, SV40 virus or genetic mutations (*BAP1, NF2, CDKN2A*) are secondary risk factors for this neoplasia [[5], [6], [7], [8], [9]].

Kelch-like (KLHL) proteins are an evolutionarily conserved family that modulates cell morphology, protein binding, dimerization and ubiquitination [10]. There are more than 40 different KLHLs identified in mammals, with KLHL19 being the most extensively studied [11,12]. Structurally, KLHL proteins have an amino-terminal Broad-complex, Tramtrack, and Bric-à-brac/poxvirus and zinc finger (BTB/POZ) domain which binds to the Cullin3 subunit of Cullin-RING ubiquitin ligase (CRL) complex, and several carboxyl-terminal Kelch repeats, which mediate substrate recognition [10,13].

Considering their roles in protein ubiquitination, KLHLs have an important impact on human physio-pathological processes including tumorigenesis and cancer progression [11]. To this regard, KLHL14 has been recently identified either as tumor promoter, in ovarian (OC) and endometrial cancers (EC) [14], or tumor suppressor, in a subtype of diffuse large B cell lymphoma (DLBCL) [15]. Therefore, the specific biological role played by KLHL14 in tumorigenesis is yet to be fully defined. Significantly, KLHL14 has been identified as a potential regulator of epithelial-mesenchymal transition (EMT) in amniotic epithelial stem cells, a cellular model where this process occurs spontaneously [16]. Notably, EMT is a hallmark of tumorigenic process and is involved in the metastatic process for the majority of solid tumors, including MM [[17], [18], [19]].

Recent works have focused on the molecular mechanisms involved in the oncogenic process in MM. A recent research supports the notion that the overexpression and mutated forms of *KRAS* lead to a more malignant and biphasic subtype of tumor [20]. We have recently demonstrated the pro-tumorigenic role of the growth factor progranulin in MM and discovered the role of EGFR and RYK pathways as functional progranulin receptors in MM [21,22]. Nevertheless, no data are available at the moment on the function of KLHL14 in MM.

Thus, the aim of this study was to characterize the role of KLHL14 in MM and decipher its potential involvement in tumorigenic processes such as cell proliferation, migration, and invasion. Here we demonstrate that KLHL14 displays nuclear expression and shuttles between the nucleus and cytoplasm in MM cells, exploiting Tumor Growth Factor-β (TGF-β)-dependent mechanisms of action. Furthermore, KLHL14 plays an anti-oncogenic role in MM by negatively regulating proliferation, migration, invasion and colony formation.

## 2 Materials and methods

### 2.1 Cell culture and treatment

Mesothelioma cell lines NCI-H2052, NCI-H2452, NCI-H28 and MSTO-211H and immortalized human mesothelial MeT-5A cells were acquired from the American Type Culture Collection (ATCC, Manassas, VA, USA), and cultured in RPMI 1640 medium (Thermo Fisher Scientific Inc, Waltham, MA, USA), supplemented with 10% FBS (R&D Systems, Minneapolis, MN, USA) and 1% Penicillin-Streptomycin (Thermo Fisher Scientific) and maintained in a 5% CO^2^ humidified incubator at 37 °C.

TGF-β1 (Transforming Growth Factor-β1 human) was purchased from Sigma-Aldrich, reconstituted according supplier's instructions and used at 10 ng/mL. To inhibit nuclear export of the protein, cells were pre-treated with Leptomycin B (MilliporeSigma and Merck KGaA, Darmstadt, Germany) at 20 nM. The *de novo* protein synthesis inhibitor Cycloheximide (C4859, Sigma-Aldrich, St. Louis, MO, USA) was resuspended in DMSO and used at a working solution of 100 μM.

The proteasomal inhibitor MG-132 (Cayman Chemical, Ann Arbor, MI, USA) was prepared by dissolving the stock in absolute Ethanol and then used at 10 μM.

### 2.2 mRNA extraction and real-time qPCR

Total RNA was extracted with Direct-zol RNA kit (Zymo research) following the manufacturer's instructions. 1 μg of total RNA was retrotranscribed using oligodT primers (Bioline, London, UK) and Tetro Reverse Transcriptase (Bioline, London, UK), following the manufacturer's instructions. qPCRs were carried out in triplicate using the SensiFAST SYBR Lo-ROX kit (Bioline London, UK) on a 7500 Fast Real-Time PCR System (Life Technologies, Carlsbad, CA, USA), with the following PCR conditions: 95 °C for 10 min, followed by 40 cycles at 95 °C for 10 s and 60 °C for 30 s. Relative quantification was performed by using the ΔΔCt method and expressed as fold change over CTR and normalized by GAPDH. Primer sequences are shown in Table 1.

**Table 1:** Primer sequences used for real-time qPCR.

**Gene**	**Forward 5′-3′**	**Reverse 5′-3′**	Tm (°C)
**KLHL14**	TGGAAGGTGCAATTACAGGTTGG	TCGAAGGTGGAGGTCCTGTC	61.3/60.9
**CDH1**	GAAAACAGCAAAGGGCTTGGA	TGGGGGCTTCATTCACATCC	59.6/60.0
**CDH2**	AGGCTTCTGGTGAAATCGCA	TGCAGTTGCTAAACTTCACATTG	59.9/58.4
**SNAI**	CCAGTGCCTCGACCACTATG	CTGCTGGAAGGTAAACTCTGGA	60.2/59.7
**TWIST**	TTCTCGGTCTGGAGGATGGA	AATGACATCTAGGTCTCCGGC	59.6/59.3
**ZEB**	GATGACCTGCCAACAGACCA	GCCCTTCCTTTCCTGTGTCA	60.0/59.9

### 2.3 siRNA transfection

Transient gene depletion was performed by siRNA strategies. NCI-H2052 and NCI-H2452 cells were transfected with either ON-TARGETplus SMARTpool Human interfering RNA (siRNAs) from Horizon Discovery (Cambridge, Cambridgeshire, UK) targeting *KLHL14* (L-023417-00-0005) or ON-TARGETplus Non-targeting Control (NTC) Pool siRNA, (D-001810–10-05), at the final concentration of 25 nM and using the DharmaFECT 1 Transfection Reagent according to supplier's protocol (Horizon Discovery, Cambridge, Cambridgeshire, UK).

24 h after transfection, cells were stimulated with TGF-β or re-seeded for migration, invasion, colony formation or proliferation assays.

### 2.4 MTS and colony formation assays

To assess proliferation, we performed MTS colorimetric assays in KLHL14-depleted and control-transfected NCI-H2052 and NCI-H2452 cells, stimulated or not with TGF-β. Briefly, 2 × 10^3^ cells/well were plated into 96-well microplates and starved for 24 h prior to TGF-β exposure. CellTiter® AQueous One Solution (Promega Corporation Madison, WI, USA) was used following supplier's guidelines. Cell growth was determined by the amount of formazan produced, analyzed by measuring the absorbance at 492 nm in a PerkinElmer VICTOR X5 2030 Multilabel plate reader (PerkinElmer, Waltham, MA, USA). The analysis was carried out at least in triplicates and the experiments performed 3 times.

For colony formation assay, 1 × 10^3^ NTC or KLHL14-depleted cells/well were seeded onto 6-well plates in complete media, allowed to attach and incubated until colonies were formed. Approximately 6–8 days after, colonies were subsequently washed with PBS, fixed with absolute methanol, and stained at room temperature for 30 min with crystal violet solution prepared at 0.5% in 25% methanol and 75% distilled water. Colonies were then additionally washed with PBS, dry and counted manually for statistical analysis.

### 2.5 Nucleus/cytoplasm fractionation

Nuclear and cytoplasmic fraction were isolated using Thermo 78833 NE-PER™ Nuclear and Cytoplasmic Extraction Reagents (Thermo Fisher Scientific) according to the manufacturer's instructions.

Briefly, adherent cells were detached and centrifuged at 500×*g* for 5 min and washed in PBS. Cell pellet was resuspended in ice-cold CER I, vortexed and incubated on ice for 10 min. Ice-cold CER II was then added to the tube, vortexed for 5 s and incubated on ice for 1 min. After, tube was vortexed for 5 more seconds and centrifuged for 5 min at ∼13,000 rpm. Supernatant containing the cytoplasmic fraction was then transferred to a new pre-chilled tube and stored at −80 °C until use.

To obtain nuclear extracts, pellet was resuspended in ice-cold NER buffer, vortexed for 15 s and placed on ice. This process was repeated by vortexing for 15 s every 10 min for a total of 40 min, then centrifuged at maximum speed for 10 min and supernatant immediately transferred to a new tube for storage at −80 °C until use. Samples were then processed for Immunoblot analysis as described below.

### 2.6 Protein extraction and western blot assay

Cells were pelleted after scraping cell dishes plated at 6–8 × 10^5^ cells/p100 dish and washing once with 1 ml of PBS 1×. Total cell lysates were then prepared by resuspending pellet with RIPA lysis buffer supplemented with 100× halt protease and phosphatase inhibitors cocktail (both from Thermo Fisher Scientific) and centrifuged at 13,000 rpm for 20 min at 4 °C recovering the supernatants at the end. Protein concentration was then measured with the Pierce™ BCA Protein Assay Kit (Thermo Fisher Scientific), and 60 μg of protein samples were run and separated on 10–12% SDS-polyacrylamide gels and subsequently blotted to nitrocellulose membranes, that were blocked with 5% non-fat milk or BSA (Bio-Rad Laboratories, Hercules, CA, USA) for 1 h at RT.

Membranes were then incubated overnight at 4 °C with the following primary antibodies diluted at 1:1000: polyclonal *anti*-KLHL14 from Invitrogen (PA-107155); monoclonal Phospho-SMAD2 (Ser465/467) (3108), SMAD 2/3 (3102), monoclonal ZO-1 (8193), monoclonal Claudin-1 XP® (13255), monoclonal SLUG (9585), monoclonal SNAIL (3879), monoclonal Vimentin XP® (46173), monoclonal *N*-cadherin XP® (13116) and Lamin A/C (2032) from Cell Signaling Technology (Danvers, MA, USA); and monoclonal GADPH (sc-365062) and monoclonal β-actin (sc-47778) from Santa Cruz Biotechnology (Dallas, TX, USA). The secondary antibodies used were mouse anti-rabbit IgG-HRP (sc-2357) and *m*-IgGk BP-HRP (sc-516102) -both from Santa Cruz Biotechnology-at 1:5000 dilution for 1 h at RT. Images were acquired using the Odyssey® XF Imaging System (LI-COR Biotechnologies, Lincoln, NE, USA), quantified with the ImageJ program and expressed as relative units (RU).

### 2.7 Immunofluorescence

For immunofluorescence analysis, cells were plated at 5 × 10^5^ cells/ml (1.5 × 10^4^ cells/well) in a 6 channel μ-Slide VI 0.4 plate (IBIDI, Gräfelfing, Bayer, Germany), washed ×3 times with PBS, fixed with 4% PFA in PBS for 15 min, washed ×3 times with PBS, permeabilized with 0.1% Triton X-100 in PBS for 10–12 min, washed ×3 times with PBS and blocked with 5% non-fat Milk in PBS for 1 h at RT. Fixed cells were incubated over-night at 4 °C with primary antibodies KLHL14 (Invitrogen, PA-107155) and/or KDEL monoclonal antibody (Enzo, 10C3) diluted 1:100 in 1% non-fat milk in PBS**.** Secondary antibodies were goat-anti Rabbit IgG (H + L) Alexa Fluor™ Plus 594 (Thermo Fisher Scientific, A32740) or goat anti-Mouse Alexa Fluor™ Plus 488 (Thermo Fisher Scientific, A32723), used for 1 h at RT in a dark place. Actin filaments were stained using the Phalloidin-iFluor 488 reagent (Abcam, Cambridge, UK), whereas nuclei were stained using the SlowFade Gold Antifade Mountant with DAPI (Thermo Fisher Scientific). Images were acquired using the Olympus cellSens program of an Olympus IX81 fluorescent microscope (Olympus, Tokyo, Japan) with a Retiga 6000 camera (QImaging, Surrey, Canada).

### 2.8 Wound healing assay

Lateral cell motility was assessed by wound healing assays. Cells were plated at a density of 2 × 10^5^ cells/well in 6-well cell culture plates, and grown for 24 h and then starved for 12 h or overnight. After PBS washings, cell monolayer was scratched using a p200 pipette tip. A third PBS wash was done to remove detached cells. Fresh SFM RPMI media supplemented or not with TGF-β was then added to each well as previously described.

Cells were monitored and Images were then taken at different time points using a DMi1 inverted microscope (Leica, Wetzlar, Germany): 0 and 48 h for NCI-H2052 cells; and 0, 24 h, 48 h, 72 h and 96 h for NCI-H2452 cells.

### 2.9 Migration and invasion transwell assays

Cell migration was assessed by transwell migration assays using 8.0 μm pore polyester membrane chambers (Corning, Glendale, AZ, USA). Serum-starved NCI-H2052 cells were seeded in SFM DMEM media supplemented with 1% P/S, 30% BSA and 50 μg/ml Transferrin in the upper chamber at a cell density of 2 × 10^4^. The lower chamber was filled with same cell media, supplemented (or not) with 10 ng/ml TGF-β for 24 h. After that, cells on the internal surface of the chamber were removed with a cotton swab, while cells on the external part were fixed with ice-cold 100% methanol for 15 min, then stained with Coomassie Brilliant Blue for 20 min and counted under a DMi1 inverted microscope (Leica, Wetzlar, Germany).

Cell invasion was assessed by using 8.0 μm pore BioCoat® Matrigel® Invasion Chambers (Corning, Glendale, AZ, USA). Experiments were performed as described for migration assays but increasing plating density to 4 × 10^4^ cells.

### 2.10 Statistical analysis

Data are presented as mean ± SEM of three single experiments (*n* = 3) with similar results. Statistical analyses were evaluated by Graph Pad Prism 8 software (RRID: SCR_002798) by applying the unpaired Student's *t*-test in a two-group comparison, and the significance of differences between groups was determined by two-way analysis of variance (ANOVA) followed by Tukey's post hoc tests for multiple comparisons at 95% confidence interval (CI). Experiments were repeated at least 3 times (*n* = 3). Statistical significance was determined at *, *p* < 0.05; **, *p* < 0.001; ***, *p* < 0.0001.

## 3 Results

### 3.1 KLHL14 expression and localization in MM cell lines

Previous studies demonstrated that KLHL14 was over-expressed in B cells [23], ovarian and endometrial cancers [14,24]. However, little is known about the role and expression of KLHL14 in MM. Thus, we initially evaluated KLHL14 expression in the three subtypes (epithelioid, biphasic and sarcomatoid) of pleural mesothelioma by interrogating cBioPortal database (Supplementary File 1). Results highlighted that KLHL14 mRNA is predominantly expressed in the epithelioid subtype (71.3 %), followed by the biphasic (26.4 %) and then by the sarcomatoid (2.3 %) subtype (Supplementary File 1). Interestingly, the presence of altered forms of this gene seems to correlate with a reduction of the overall survival rate but the number of reported cases is still insufficient to determine the statistical significance of this data (Supplementary File 1).

**Fig. 1:**
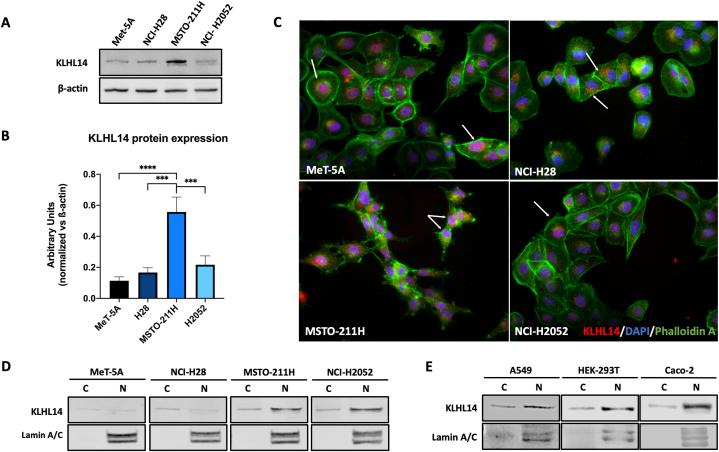
**KLHL14 expression and localization in cancer cells**. A, B) KLHL14 protein levels in MM cell lines (NCI-H28, MSTO-211H, NCI-H2052) and immortalized mesothelial cell line (Met-5A) as assessed by western immunoblot with specific antibodies. C) Immunofluorescence of KLHL14 (red), DAPI (blue) and Phalloidin A (green). D, E) KLHL14 nucleocytoplasmic distribution in MM cell lines (D) and other cancer cell lines (E) was determined by fractionation. Statistical analysis was carried out by using the unpaired Student's *t*-test: ***p < 0.001, ****p < 0.0001. Uncropped gels and blots are in Supplementary Fig. 2. (For interpretation of the references to color in this figure legend, the reader is referred to the Web version of this article.)

Therefore, we then decided to investigate by immunoblot KLHL14 expression levels in a panel of MM cell lines representing all histological subtypes: epithelioid (NCI-H28), sarcomatoid (NCI-H2052) and biphasic (MSTO-211H), using the immortalized human mesothelial cell line Met-5A as control. KLHL14 was expressed at higher levels in MSTO-211H compared to the other cell lines (Fig. 1A and B). We then investigated KLHL14 expression by immunofluorescence analysis (Fig. 1C). Contrary to previously published data, which demonstrated cytoplasmic localization of KLHL14 [25] all MM cell lines showed nuclear positivity for KLHL14 (Fig. 1C), highlighting a prevalent nuclear expression in Met-5A, MSTO-211H and NCI-H2052 cells. However, we noticed a remarkable difference in NCI-H28 cells, in which KLHL14 localized in both the nucleus and cytoplasm.

To more specifically analyze the cellular distribution of KLHL14, we then isolated nuclear and cytoplasmic fractions of the various mesothelial and MM cells. While NCI-H2052 cells and MSTO-211H showed high nuclear positivity for KLHL14, in NCI-H28 and Met-5A cells KLHL14 was mostly expressed in the cytoplasmic fraction, with low nuclear localization (Fig. 1D). Notably, KLHL14 prevalently localized within the nucleus of NCI-H2052 cells (Fig. 1C and D). Finally, we demonstrated that in other cancer cells lines (A549, Caco-2 and HEK-293T), KLHL14 showed both cytoplasmic and nuclear expression, with prevalent nuclear localization observed in HEK-293T and Caco-2 cells too (Fig. 1E).

### 3.2 Bioinformatic analyses hint KLHL14 nuclear localization

In order to understand the mechanisms of nuclear localization of KLHL14, we first looked for classical nuclear localization (cNLS) or nuclear export signals (NES) within the protein sequence using NLStradamus [26] and NLSdb [27] bioinformatic tools. However, in agreement with a previous study [25], these tools failed to identify any cNLS or NES, therefore suggesting that different signals or cellular mechanisms regulate KLHL14 translocation into the nucleus.

### 3.3 *KLHL14* transient depletion upregulates EMT-related factors

**Fig. 2:**
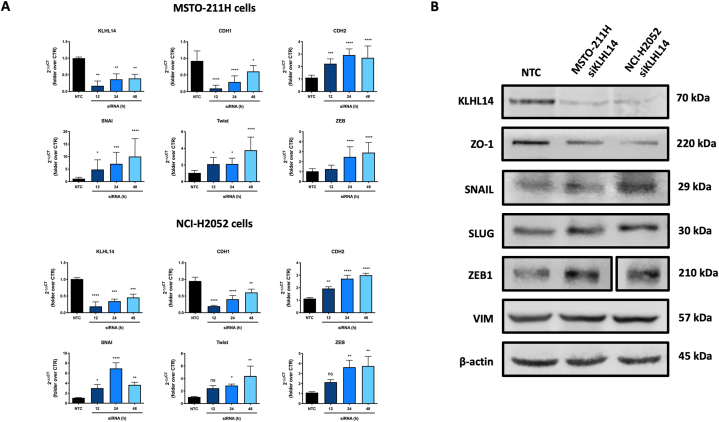
**KLHL14 affects EMT-related factors**. A) Real-time qPCR showing mRNA levels of ZEB, SNAI, Twist, CHD2 and CHD1 in MSTO-211H and NCI-H2052 cells. B) Representative western immunoblots for EMT pathway-related proteins in MSTO-211H and NCI-H2052 cells. *p < 0.05, **p < 0.01, ***p < 0.001, ****p < 0.0001. Uncropped gels and blots are in Supplementary Fig. 3.

*KLHL14* has been recently identified as a gene possibly involved in epithelial-mesenchymal transition (EMT) [16] For this reason, we first evaluated the effects of transient KLHL14 depletion on EMT-related genes and protein expression, using MSTO-211H and NCI-H2052 cell lines (Fig. 2). As a result, downregulation of KLHL14 with specific siRNAs induced a significant increase in mRNAs for EMT-transcription factors (EMT-TFs) *ZEB*, *SNAI* and *Twist* (Fig. 2A). Conversely, *KLHL14* transient depletion induced upregulation of mesenchymal gene *CHD2* (*N*-Cadherin) while significantly reduced the expression of the epithelial marker *CHD1* (*E*-Cadherin) (Fig. 2A). These results were further confirmed by analyzing EMT-related protein expression levels by Western Blotting (Fig. 2B). To this regard, transient depletion of *KLHL14* induced upregulation of the EMT-TFs SNAIL, SLUG and ZEB1 while it reduced the expression of the epithelial marker ZO-1 (Fig. 2B). However, at a protein level, no significant differences were observed in the expression of the late mesenchymal marker vimentin between control and *KLHL14*-depleted cells (Fig. 2B). These results suggest that KLHL14 may have a role in regulating the formation of intermediate Epithelial/Mesenchymal (E/M) phenotypes [28]. For this reason, we performed further studies to better understand the role of KLHL14 in MM.

### 3.4 KLHL14 translocates from nucleus to cytoplasm in response to TGF-β

**Fig. 3:**
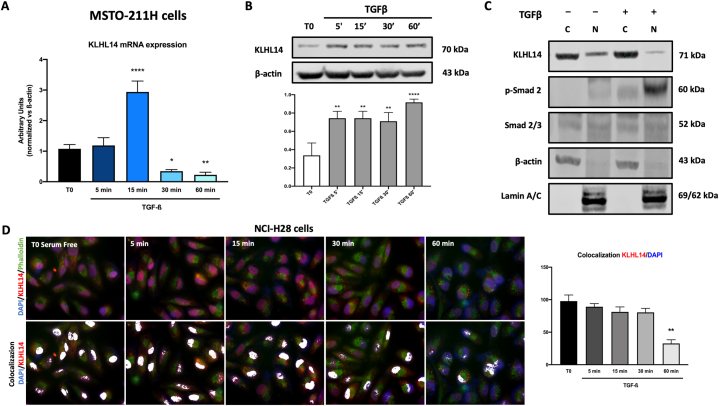
**TGF-β stabilizes KLHL14 expression and increases its stability**. A, B) KLHL14 mRNA levels were assessed by qt-PCR (A) and immunoblot (B) in MSTO-211H. C) Immunoblot analysis of nuclear and cytoplasmic levels of *p*-SMAD 2/3 in MSTO-211H cells. D) Immunofluorescence analysis of KLHL14 (red), DAPI (blue) and Phalloidin A (green) in NCI-H28 with quantification of KLHL14 and DAPI colocalization signals. White signals represent nuclear localization of KLHL14, where red and blue dyes colocalize **p < 0.01, ****p < 0.0001. Uncropped gels and blots are in Supplementary Fig. 4. (For interpretation of the references to color in this figure legend, the reader is referred to the Web version of this article.)

In order to further define the role of KLHL14 in the modulation of EMT-related proteins, we explored whether TGF-β, an important EMT inducer [29,30], could affect KLHL14 levels. Notably, TGF-β induced a time-dependent upregulation of KLHL14 mRNA and protein expression levels (Fig. 3A and B). The activation of TGF-β signaling pathway was assessed by measuring phosphorylation and nuclear translocation of Smad 2/3, the intracellular signal transducer and transcriptional modulator activated by TGF-β (Fig. 3C). Significantly, we found that TGF-β stimulation induced a decrease in nuclear expression of KLHL14, while slightly increasing cytoplasmic accumulation of the protein (Fig. 3C). Based on these results, we then evaluated nuclear and cytoplasmic expression of KLHL14 by immunofluorescence analysis at different time points of TGF-β stimulation (5, 15, 30 and 60 min), using DAPI as nuclear staining and phalloidin as marker of the cytoplasmic compartment (Fig. 3D). Interestingly, TGF-β stimulation induced a time-dependent decrease in nuclear localization of KLHL14, which was significantly exported from the nucleus after 60 min (Fig. 3D).

**Fig. 4:**
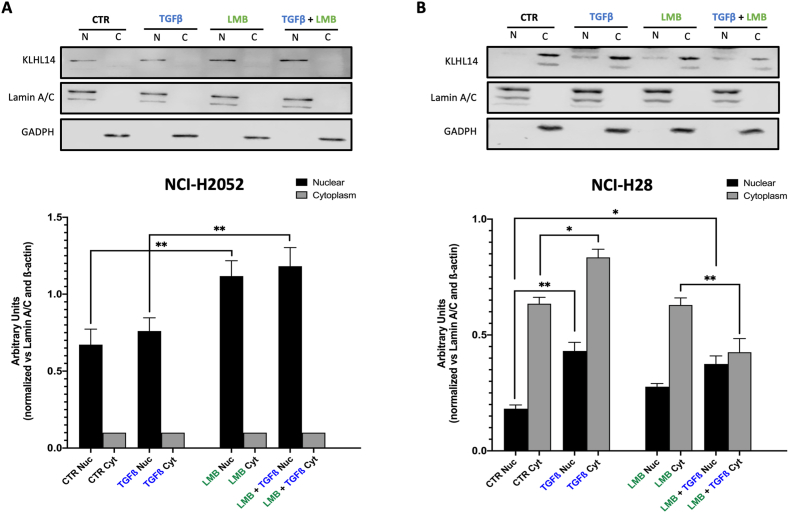
**TGF-β induces nuclear-cytoplasmic shuttling of KLHL14**. A, B) Immunoblots and densitometric analyses of KLHL14 nuclear and cytoplasmic levels obtained by fractionation of cell lysates derived from NCI-H2052 (A) and NCI-H28 (B) cells pre-treated or not with 20 nM Leptomycin B (LMB) and/or TGF-β. *p < 0.05, **p < 0.01. Uncropped gels and blots are in Supplementary Fig. 5.

In order to assess whether the reduction of KLHL14 in the nuclear fraction was associated with cytoplasmic translocation, we used two different cell lines with different KLHL14 localization: NCI-H28 cells, where KLHL14 localized in both compartments but displayed higher expression in the cytoplasm, and NCI-H2052 cells, where KLHL14 was prevalently expressed in the nuclear fraction. Both cell lines were exposed to Leptomycin B (LMB), a nuclear export inhibitor, and then treated or not with TGF-β (Fig. 4). In NCI-H2052, TGF-β stimulation only induced a slight increase of KLHL14 nuclear expression compared to control cells (CTR) (Fig. 4A). Conversely, pre-treatment with LMB, alone or in combination with TGF-β, promoted a significant accumulation of nuclear KLHL14 compared to CTR or TGF-β alone. However, the effects of TGF-β on KLHL14 cytoplasmic fraction were much more significant in NCI-H28 cells (Fig. 4B). In fact, in NCI-H28 cells stimulated with TGF-β we noticed a significant increase in nuclear expression and cytoplasmic accumulation of KLHL14 (Fig. 4B). On the other hand, pre-treatment with LMB alone slightly increased the nuclear fraction of KLHL14 without changing its cytoplasmic expression. However, the combination of LMB and TGF-β significantly increased KLHL14 nuclear pool without inducing any cytoplasmic accumulation (Fig. 4B). Overall, these results demonstrate that TGF-β promotes KLHL14 upregulation in a time-dependent manner. Moreover, TGF-β stimulation may influence KLHL14 nuclear-cytoplasmic translocation in a manner which is dependent on the MM cell histological subtype.

### 3.5 TGF-β stimulates *de novo* KLHL14 synthesis and increases its stability

**Fig. 5:**
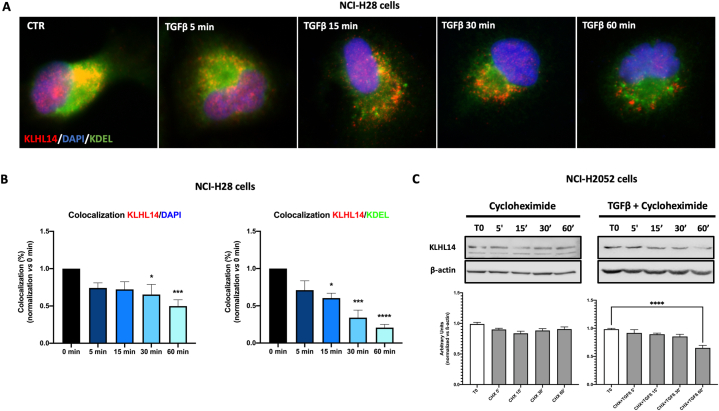
**TGF-β affects KLHL14 de novo protein synthesis**. A, B) Immunofluorescence and colocalization analysis of KLHL14 (red) and the ER-resident proteins specific dye KDEL (green) in NCI-H28 cells pre-treated with Cycloheximide (CHX) and then stimulated with TGF-β for the indicated time points (0–60′). CTR: unstimulated cells. C) Immunoblots of KLHL14 protein levels in the presence of 100 μM CHX, followed or not by TGF-β treatment of NCI-H2052 cells. *p < 0.05, ***p < 0.001, ****p < 0.0001. Uncropped gels and blots are in Supplementary Fig. 6. (For interpretation of the references to color in this figure legend, the reader is referred to the Web version of this article.)

To better understand how TGF-β modulates KLHL14 stability, we blocked *de novo* protein synthesis with cycloheximide (CHX), which interferes with the translocation step during elongation. To this aim, MSTO-211H and NCI-H28 cells were exposed to CHX from 0 to 60 min, both treated or not with TGF-β, and KLHL14 localization and expression were evaluated by immunofluorescence analysis and immunoblot assays (Fig. 5). Notably, as KLHL14 was originally described as localizing in the endoplasmic reticulum (ER) [25], we performed the following experiments by staining nuclei with DAPI and ER with KDEL-specific dye, which recognizes a sequence essential for maintaining resident proteins in the ER lumen, reducing their secretion [31]. Intriguingly, the addition of TGFβ to CHX significantly reduced KLHL14 localization not only into nucleus but also in ER, in a time-dependent manner (Fig. 5A and B). Moreover, differently from the treatment with TGF-β alone (Fig. 3B), the combination of TGF-β and CHX significantly reduced the expression of KLHL14 in a time-dependent manner (Fig. 5C). Interestingly, pre-treatment with CHX only did not induce any remarkable changes in KLHL14 protein expression over the time (Fig. 5C), making us conclude that TGFβ increases KLHL14 expression in a time-dependent manner by stimulating *de novo* synthesis of KLHL14 protein in MM cells.

**Fig. 6:**
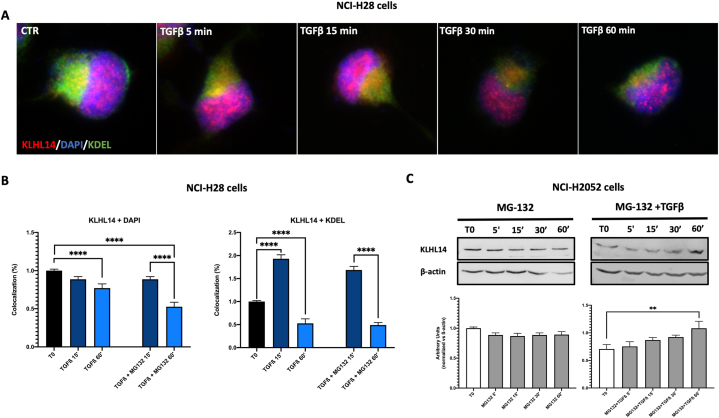
**KLHL14 proteasomal degradation analysis**. A, B) Immunofluorescence and colocalization analysis of KLHL14 (red) and KDEL (green) in NCI-H28 cells pre-treated with MG-132 and then stimulated or not (CTR) with TGF-β at the indicated time points (0–60 min). C) Immunoblots of KLHL14 protein levels in the presence of 10 μM MG-132, followed or not by TGF-β treatment in NCI-H2052 cells. **p < 0.01, ****p < 0.0001. Uncropped gels and blots are in Supplementary Fig. 7. (For interpretation of the references to color in this figure legend, the reader is referred to the Web version of this article.)

Since TGF-β affected KLHL14 stability, we then evaluated whether KLHL14 downregulation was dependent on proteasomal degradation. Thus, we measured KLHL14 stability in the presence of MG-132, a proteasome inhibitor [32,33] in either unstimulated (CTR) or TGF-β-treated MM cells (Fig. 6). We first assessed subcellular localization of KLHL14 after TGF-β stimulation in the presence of MG-132 using DAPI and KDEL markers for nuclear and ER staining, respectively (Fig. 6A). Although nuclear localization of KLHL14 was significantly reduced, we reported a relevant increase of its ER localization occurring only after 15 min of TGF-β stimulation (Fig. 6B). On the other hand, KLHL14 protein was stabilized by MG-132 (Fig. 6C). In fact, pre-treatment with MG-132+TGF-β significantly increased KLHL14 protein expression after 1 h compared to CTR (Fig. 6C). Conversely, KLHL14 levels remained unaffected over time when cells were exposed to MG-132 alone (Fig. 6C). In summary, these results suggest that TGF-β reduces KLHL14 stability mainly by affecting *de novo* protein synthesis but without influencing its proteasomal degradation.

### 3.6 KLHL14 depletion enhances cell proliferation, migration and invasion in MM *in vitro* models

**Fig. 7:**
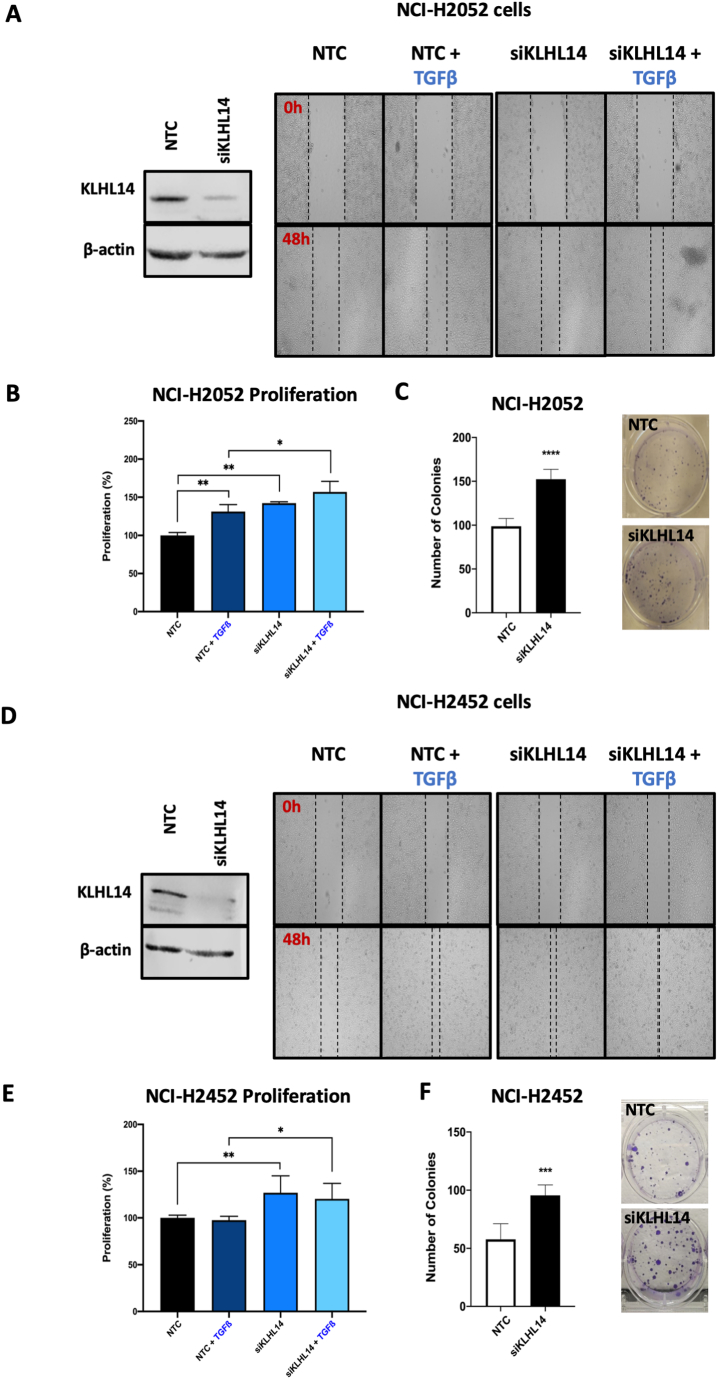
**KLHL14 affects lateral motility, viability and colony formation of MM cells**. Wound healing assay of (A) NCI-H2052 and NCI-H2452 (D) with representative immunoblots of KLHL14 levels after transient silencing by siRNA approaches after TGF-β stimulation or not. MTS proliferation assay of NCI-H2052 (B), and NCI-H2452 (E) cells under the same conditions. Graphics show data expressed as a percent of viable cells, considering NTC controls as 100% viability. Long-term colony formation assays performed in NCI-H2052 (C) and NCI-H2452 cells (F), treated or not with TGF-β. Abbreviation: Non-targeting control siRNA (NTC); KLHL14-transiently silenced cells (siKLHL14). *p < 0.05, **p < 0.01, ****p < 0.0001. Uncropped gels and blots are in Supplementary Fig. 8.

In order to investigate the biological functions of KLHL14 in response to TGF-β, we initially tested lateral motility of MM cells comparing controls (non-targeting control, NTC) with cells transiently depleted of *KLHL14* (siKLHL14). We first silenced cultured cells and then performed wound healing assays using sarcomatoid NCI-H2052 (Fig. 7A, B and C) and epithelioid NCI-H2452 (Fig. 7D, E and F), exposed or not to TGF-β. The results of these experiments showed that TGF-β promoted lateral motility of MM cells (Fig. 7A and D). Notably, KLHL14 transient depletion led to a significant enhancement of TGF-β-induced wound closure in both NCI-H2052 (Fig. 7A) and NCI-H2452 cells (Fig. 7D) as compared to NTC transfected control cells.

We then investigated the effects of TGF-β stimulation on proliferation of NCI-H2052 (Fig. 7B) and NCI-H2452 cells (Fig. 7E) after KLHL14 transient depletion. TGF-β significantly increased cell proliferation of only NCI-H2052 cells (Fig. 7B) without affecting the growth rate of NCI-H2452 cells (Fig. 7E). However, transient depletion of KLHL14 significantly enhanced cell proliferation of both cell lines compared to NTC controls (Fig. 7B and E).

Furthermore, we evaluated the ability of colony formation in monolayer of both NCI-H2052 (Fig. 7C) and NCI-H2452 (Fig. 7F) cells following transient depletion of KLHL14. As a result, we observed higher number and larger size of colonies formed in KLHL14-depleted cells compared to NTC controls in both cell lines (Fig. 7C and F).

**Fig. 8:**
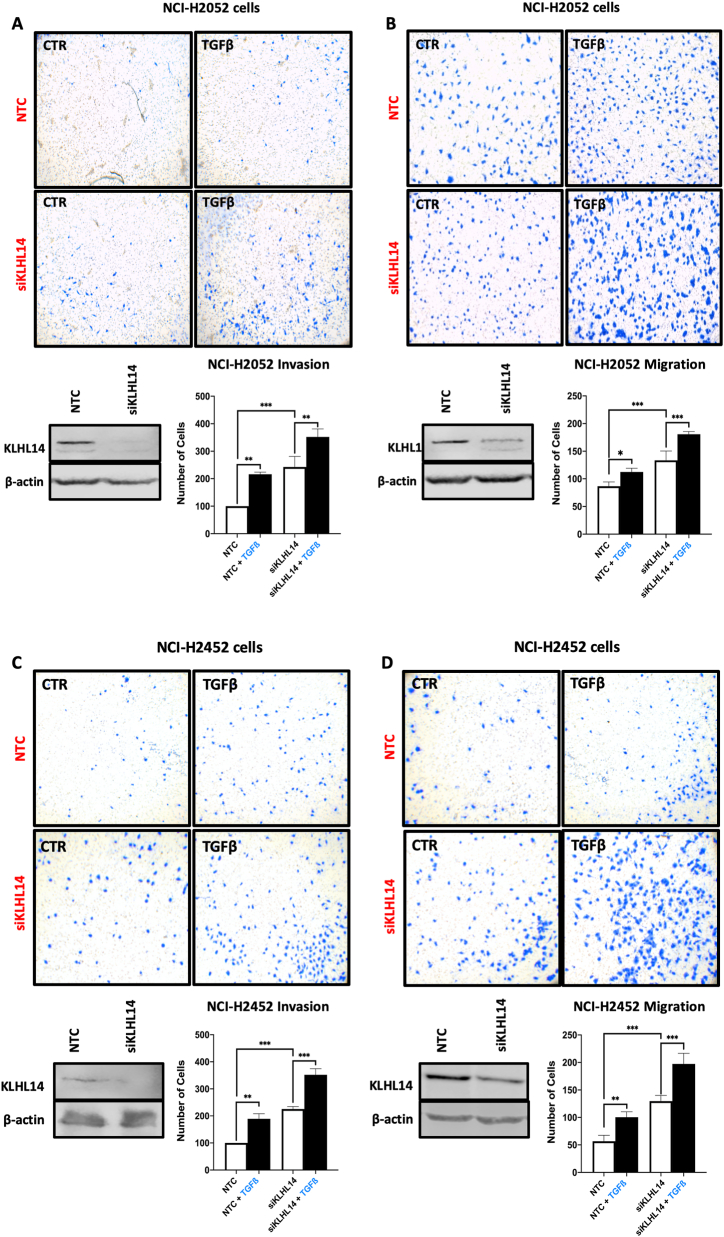
**KLHL14 depletion enhances migration and invasion in NCI-H2052 and NCI-H2452 cells**. A, B) Migration and Invasion assays of NCI-H2052 control cells transfected NTC or KLHL14-transiently silenced (siKLHL14), in presence or not (CTR) of TGF-β. C, D) Migration and invasion assays of NCI-H2452 control cells transfected NTC or siKLHL14, in presence or not (CTR) of TGF-β. Abbreviation: Non-targeting control siRNA (NTC). *p < 0.05, **p < 0.01, ***p < 0.001. Uncropped gels and blots are in Supplementary Fig. 8.

Lastly, we evaluated cell migration in transwells and invasion through Matrigel using NCI-H2052 and NCI-H2452 cells (Fig. 8). In agreement with previous results, TGF-β significantly increased both migration (Fig. 8A and C) and invasion through Matrigel (Fig. 8B and D) of both NTC-transfected cells lines compared to the relative unstimulated controls (CTR). Interestingly, transient depletion of KLHL14 (siKLHL14) further enhanced migration (Fig. 8A and C) and invasion (Fig. 8B and D) of both unstimulated (CTR) and TGF-β-stimulated cells. Overall, these results indicated that KLHL14 may play an important role in modulating cell proliferation and motility and suggested for the first time an anti-oncogenic role of KLHL14 in MM cells.

## 4 Discussion

MM is an aggressive cancer characterized by poor prognosis, low survival rates, and a limited effective therapeutic strategy [1,4]. Here, we describe a novel KLHL14 anti-oncogenic action in MM, and demonstrated that KLHL14 inhibition enhanced cell proliferation, migration, invasion, colony formation and EMT. Moreover, for the first time, we show nuclear localization of KLHL14 and elucidated a novel mechanism of action involving KLHL14 nucleocytoplasmic shuttling in response to TGF-β stimulation.

KLHL14 is a poorly characterized protein belonging to the Kelch-like family, which is involved in the regulation of cell morphology and ubiquitination, and plays a crucial role in disease and tumor development [10]. To this regard, through a combination of RNA-seq and network analyses, Di Lollo V. and Canciello A. (2020) had first demonstrated that KLHL14 played a role in controlling EMT in Amniotic Epithelial Cells (AECs), a physiological cell model of EMT [16]. Indeed, AECs are a subset of placental stem cells that spontaneously undergo EMT upon *in vitro* amplification, but this process can be easily inhibited or reverted by adding progesterone [34,35]. Notably, in their study *KLHL14* was identified among the genes predicted to play an important role in maintaining AECs epithelial phenotype and preventing their mesenchymal shift [16]. In the present study, we evaluated the role of KLHL14 in EMT in a pathological model of EMT, such as the one occurring in MM cells. In this respect, EMT represents one of the molecular mechanisms associated with MM onset and invasiveness [17,36]. Indeed, it has been demonstrated how asbestos exposure increases TGF-β levels, which in turn promote a malignant phenotype and invasiveness via EMT events [17]. In addition, as reported by Ramesh et al., the inhibition of EMT represents a potential therapeutic approach against MM, further highlighting the central role of this process in MM [37]. Accordingly, here we demonstrate that KLHL14 depletion induces an upregulation of EMT transcription factors (EMT-TFs) expression with a consequent downregulation of epithelial markers. Therefore, in agreement with previous findings [16], these results confirm that KLHL14 is essential to prevent EMT, even in absence of TGF-β stimulation.

Interestingly, KLHL14 displayed different expression and subcellular distribution in cells derived from different MM subtypes. In fact, although all cell lines showed nuclear and cytoplasmic expression, KLHL14 subcellular localization changed depending on the MM phenotype. On one hand, cytoplasmic expression was prevalent in MM epithelial subtypes whereas nuclear expression increased and was predominant in mixed and sarcomatoid subtype. These results suggest that KLHL14 subcellular localization may be related to cell phenotype, further pointing out an association between this protein and the EMT process.

To the best of our knowledge, this represents the first work to report nuclear expression of KLHL14. In fact, Giles and colleagues (2009), who first identified KLHL14 as protein interacting with TorsinA (Printor), demonstrated KLHL14 localization mainly in the ER and, to a lesser extent, in the nuclear envelope (NE) [25]. Subsequently, other research groups analyzed KLHL14 functions in different cancer and non-cancer cells, in particular B cells and neurons [14,15,23,24,38]. Notably, in agreement with other research groups, we did not identify a canonical nuclear localization sequence (NLS). However, KLHL14 is not the only member of its family displaying nuclear expression without possessing a canonical NLS, as it is the case for KLHL37 [39]. Furthermore, differently from any other BTB-containing proteins, the BTB domain of KLHL14 is interrupted by an insertion of a 48 amino acid which forms a proline/glutamine (P/Q)-rich sequence [25]. Significantly, such particular clustered arrangement of proline and glutamine is specifically found within the activation domain of transcription factors [40], suggesting that this sequence could be, at least in part, contributing to the nuclear positivity for KLHL14.

Here, we also demonstrate that KLHL14 expression and localization are modulated by TGF-β stimulation. In particular, although TGF-β initially upregulates KLHL14 expression, it induces a time-dependent shuttling of the protein from the nucleus to the cytoplasmic compartment. As Cullin3-based ubiquitin E3 ligase, KLHL14 proteins assemble homodimers through their BTB domains, recognize protein substrates through their Kelch beta-propeller repeats and recruit E2 ubiquitin-conjugating enzyme through their association with Cullin3 [41]. To this regard, *KLHL14* was recently identified as tumor suppressor gene which is recurrently mutated in mature B cell malignancies [23]. In this work, Choi J. and colleagues (2020), demonstrated that KLHL14 binds Cullin3 and act as ubiquitin ligase by binding and downregulating the expression of B-cell receptor. Therefore, identifying the specific protein substrate/s of KLHL14 is crucial to understand the specific mechanism of action and its involvement in the EMT process. Indeed, KLHL14 translocation to the cytoplasm upon TGF-β stimulation might have a dual meaning: in this scenario KLHL14 may regulate a pro-EMT substrate in the nucleus and TGF-β stimulation may foster its release by inducing KLHL14 translocation into cytosol, thereby triggering EMT. Alternatively, KLHL14 may recognize an *anti*-EMT substrate in the cytosol upon its cytosol translocation stimulated by TGF-β, triggering therefore EMT. Both the hypotheses could explain the *anti*-EMT role of KLHL14, whereas the TGF-β-induced KLHL14 upregulation could be explained as inhibitory feedback control over the EMT process. However, further experiments are needed to assess the specific mechanism of action of KLHL14 in MM.

In this study, we highlighted a strict association of KLHL14 with TGF-β signaling. In fact, KLHL14 levels are sensitive to TGF-β stimulation, which also increases KLHL14 stability by affecting its *de novo* synthesis other than its proteasomal degradation. However, whilst this study mainly explores the effect of TGF-β signaling and EMT-related factors in regulating KLHL14 expression in MM, additional experiments are presently ongoing to investigate whether other potential mechanisms may also be involved.

In addition, we also demonstrate the anti-oncogenic role of KLHL14 in MM. Indeed, *KLHL14* gene depletion enhanced cell proliferation, lateral motility of both sarcomatoid and epithelioid MM cells, and promoted migration and invasion through Matrigel of NCI-H2052 cells. Similar results were observed in colony-forming activity as in fact downregulation of KLHL14 expression increased the number and size of colonies formed by MM cells. Intriguingly, our results support the hypothesis that KLHL14 can act as a suppressor of TGF-β-mediated transforming activity in MM.

Actually, there is very limited literature on KLHL14 action and its association with TGF-β/EMT signaling pathway, but data on other KLHL proteins have demonstrated correlations with a variety of cellular processes involved in tumor development and metastasis [11,13]. Two manuscripts reported an association between KLHL14 and increased tumor proliferation and migration in ovarian and endometrial cancer [24,42]. These results differ from our findings but this is likely due to the different tumor type and cell model used, as well as the type of analyses conducted, mainly based on predictive bioinformatic models.

Altogether, the present study provides the novel observation of a potential mechanistic role of TGF-β signaling in the regulation of KLHL14 expression, nuclear translocation and stability. In addition, our results support the fact that KLHL14 may function as a tumor suppressor in MM. Additional researches focused on understanding the precise mechanism of action of KLHL14/TGF-β axis are still needed, including *in vivo* experiments and future clinical trials, to potentially develop KLHL14-based drugs that can be later be applied in MM and other deadly cancers with low therapeutic options.

## Data availability statement

The data that support the findings of this study are available upon reasonable request from the corresponding authors.

## CRediT authorship contribution statement

**Angelo Canciello:** Writing – review & editing, Writing – original draft, Visualization, Methodology, Investigation, Data curation, Conceptualization. **Reyes Benot Domínguez:** Writing – review & editing, Writing – original draft, Methodology, Investigation, Data curation, Conceptualization. **Barbara Barboni:** Funding acquisition, Conceptualization. **Antonio Giordano:** Resources, Funding acquisition. **Andrea Morrione:** Writing – review & editing, Supervision, Methodology, Funding acquisition, Conceptualization.

## Declaration of competing interest

The authors declare that they have no known competing financial interests or personal relationships that could have appeared to influence the work reported in this paper.
