# Supplementary material for: Characterization of KLHL14 anti-oncogenic action in malignant mesothelioma
Source: Heliyon. 2024 Mar 9;10(6):e27731. doi: 10.1016/j.heliyon.2024.e27731 (PMC10950656; doi:10.1016/j.heliyon.2024.e27731)
Supplement: Multimedia component 1 [file mmc1.pdf]

A

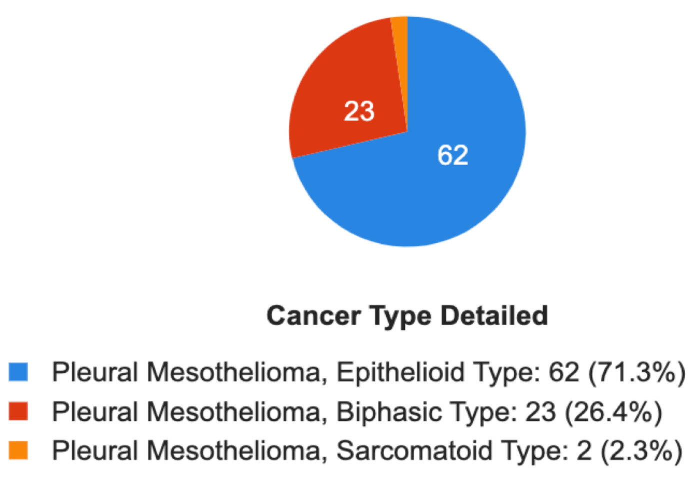

B

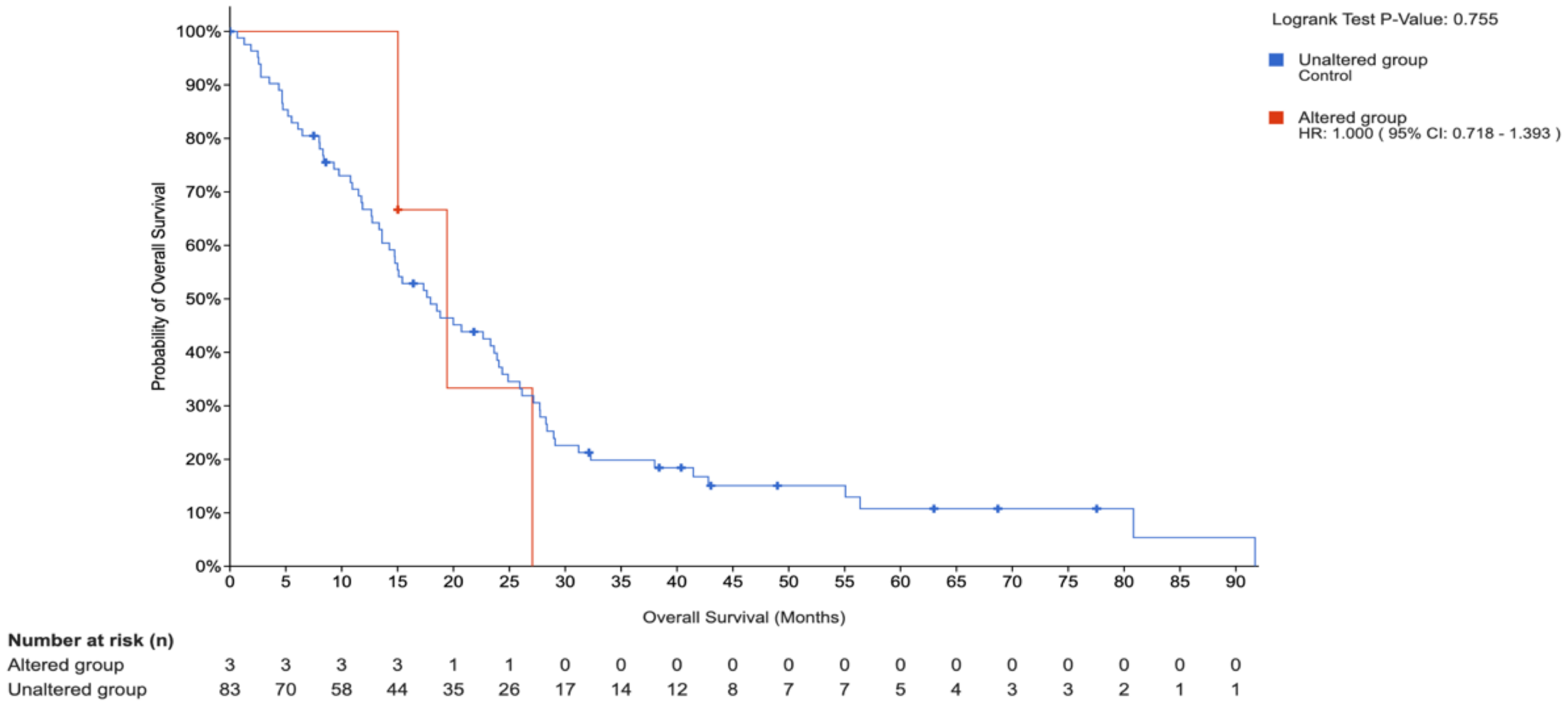

Supplementary File 1. KLHL14 expression in the epithelioid, biphasic and sarcomatoid subtypes of pleural mesothelioma in cBioPortal database. A) KLHL14 mRNA is predominantly expressed in the epithelioid subtype (71.3 %), followed by the biphasic (26.4 %) and then by the sarcomatoid (2.3 %) subtype (Supplementary File 1). B) The presence of altered forms of KLHL14 seems to correlate with a reduction of the overall survival rate but the number of reported cases is still insufficient to determine the statistical significance of this data. Database: Mesothelioma (TCGA, PanCancer Atlas), 87 Samples.
