# Supplementary figures and images for: Characterization of KLHL14 anti-oncogenic action in malignant mesothelioma
Source: Heliyon. 2024 Mar 9;10(6):e27731. doi: 10.1016/j.heliyon.2024.e27731 (PMC10950656; doi:10.1016/j.heliyon.2024.e27731)

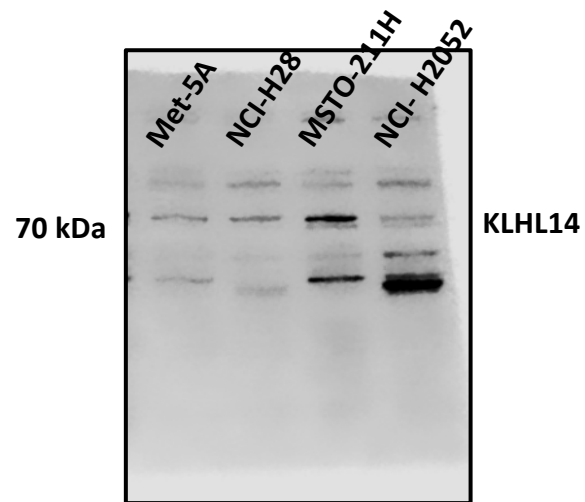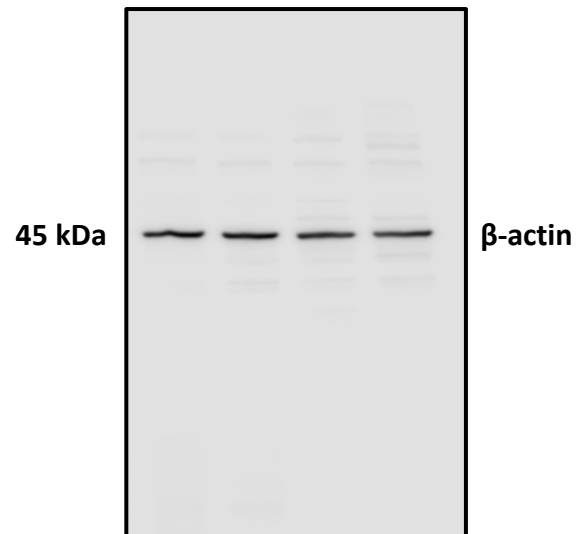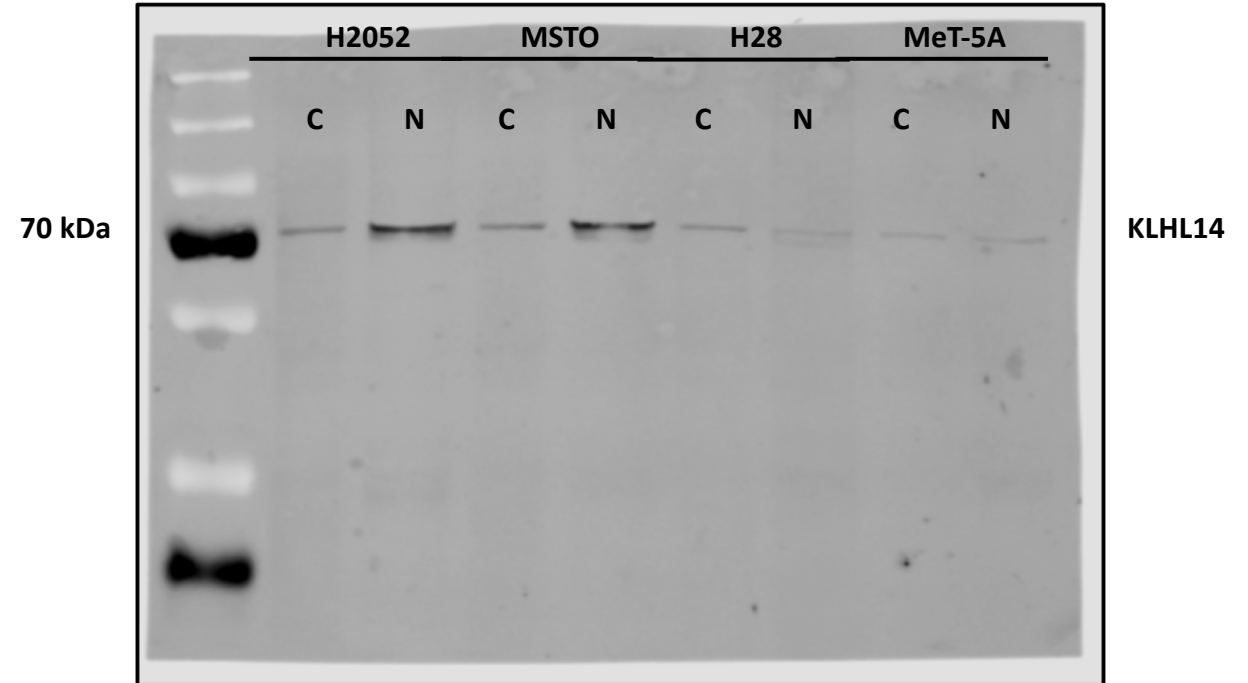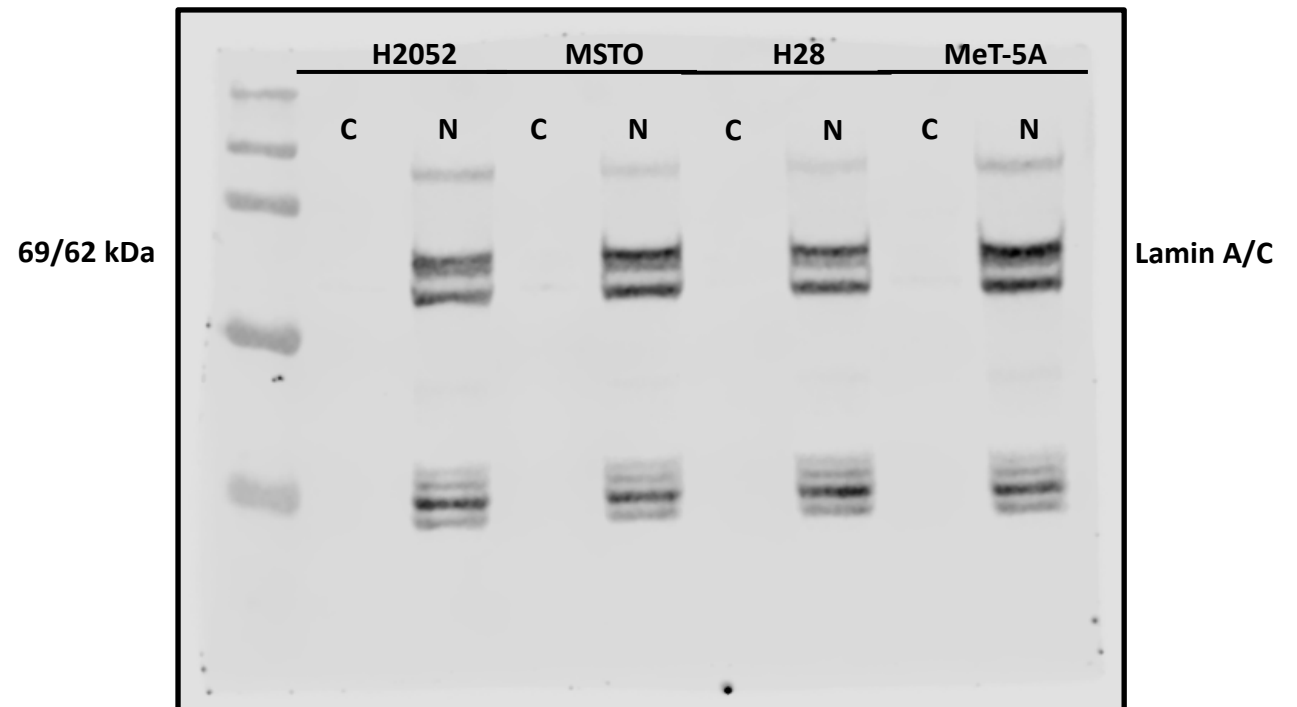

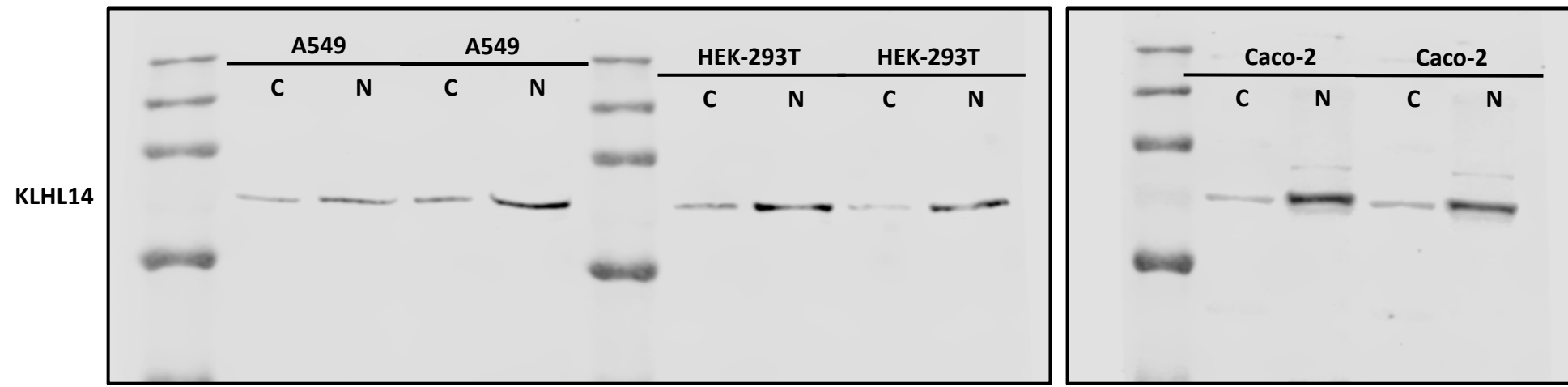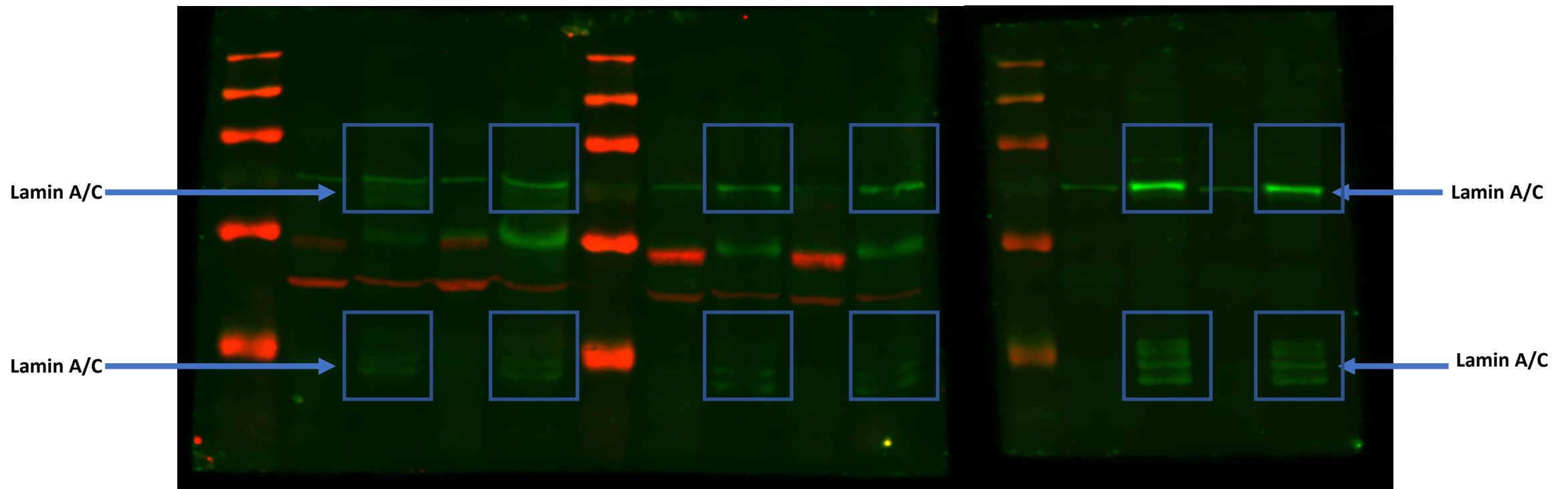

Supplement: Multimedia component 2 [file mmc2.pdf]

EMT

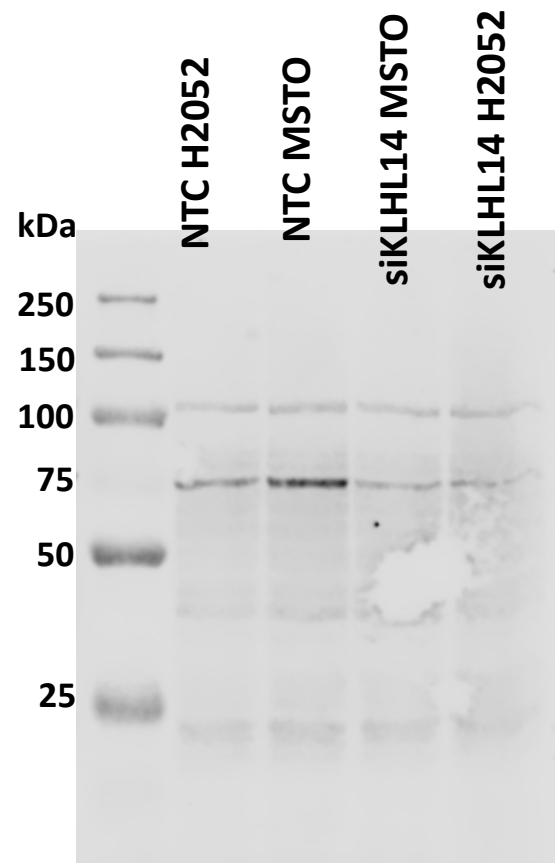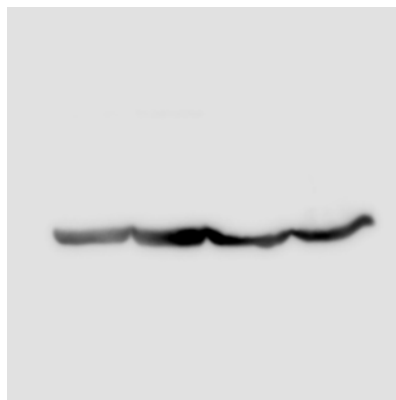

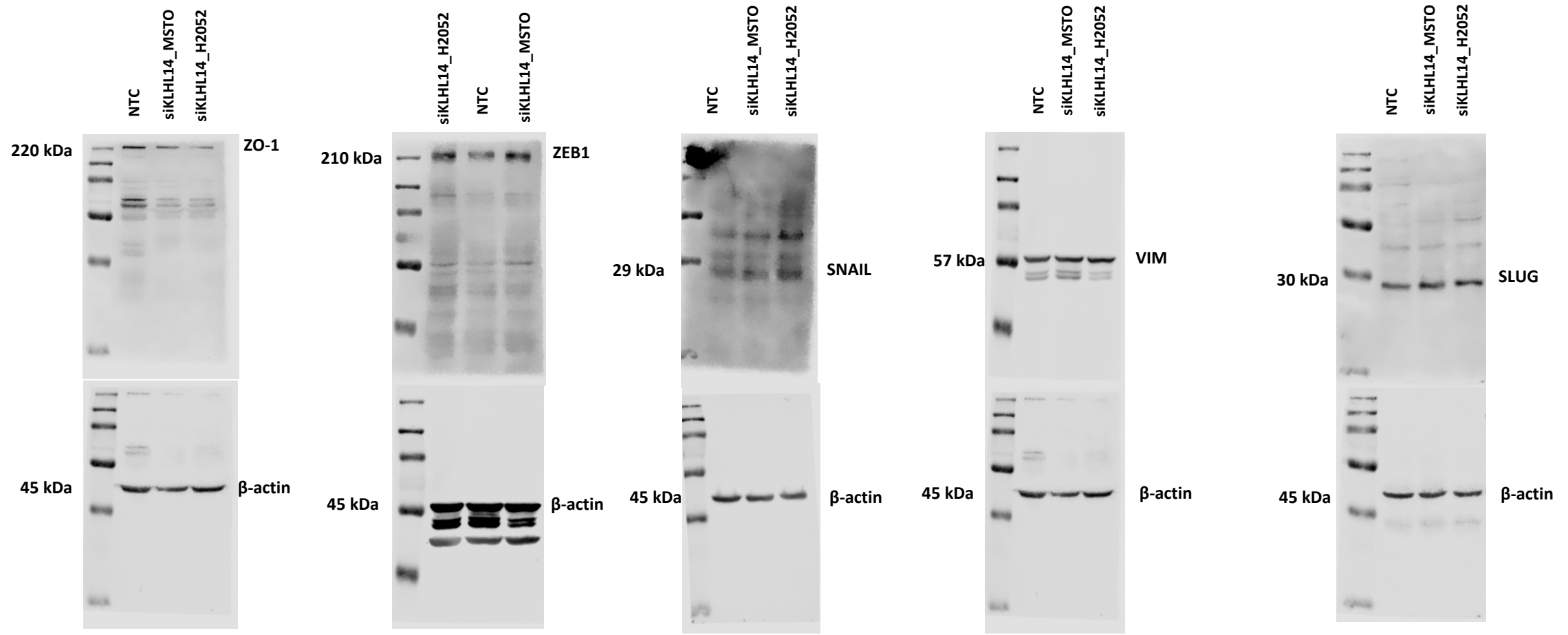

Supplement: Multimedia component 3 [file mmc3.pdf]

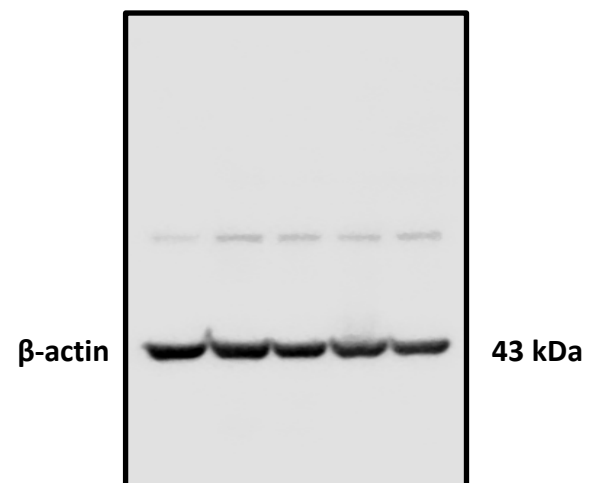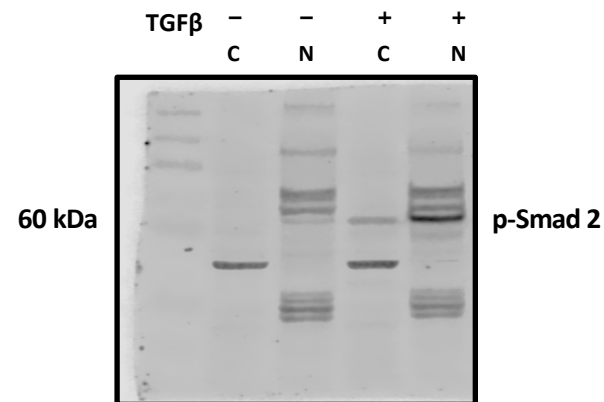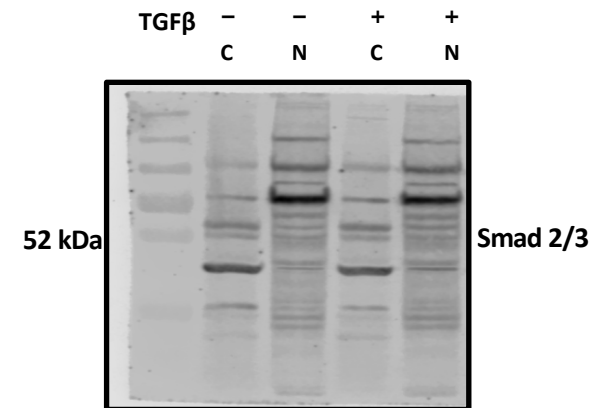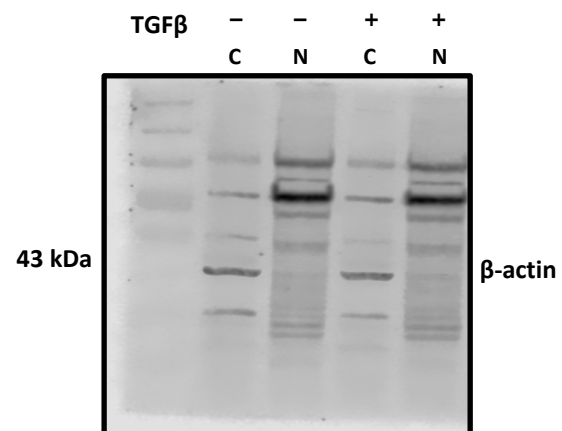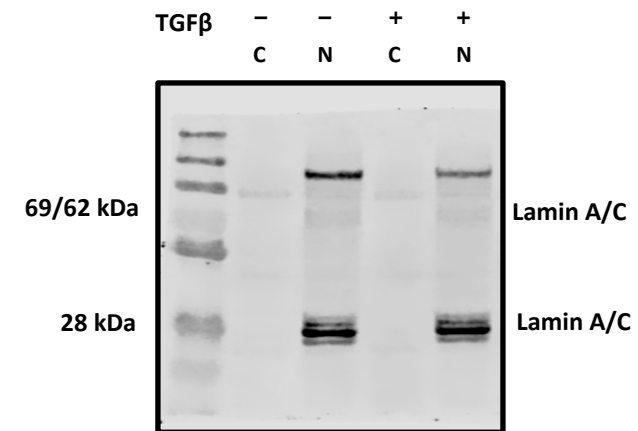

Supplement: Multimedia component 4 [file mmc4.pdf]

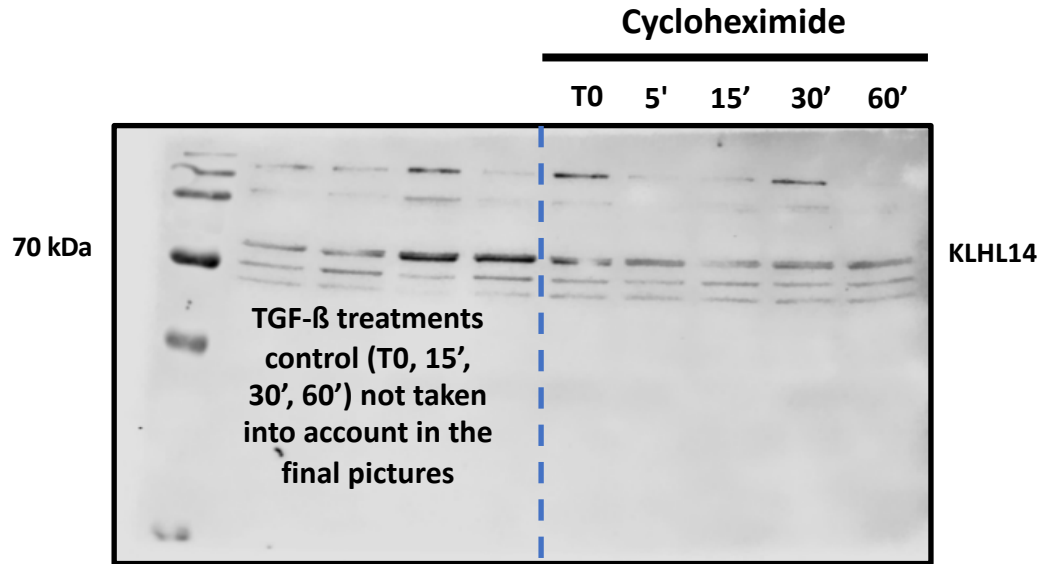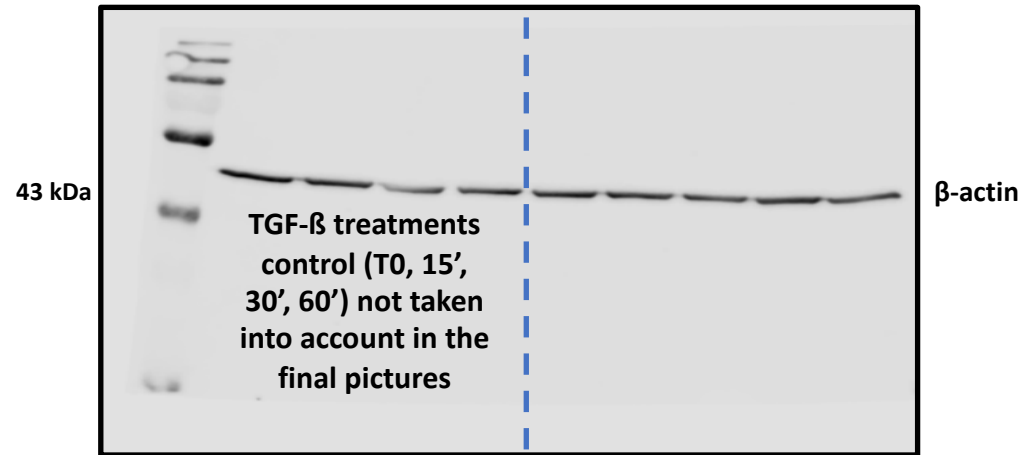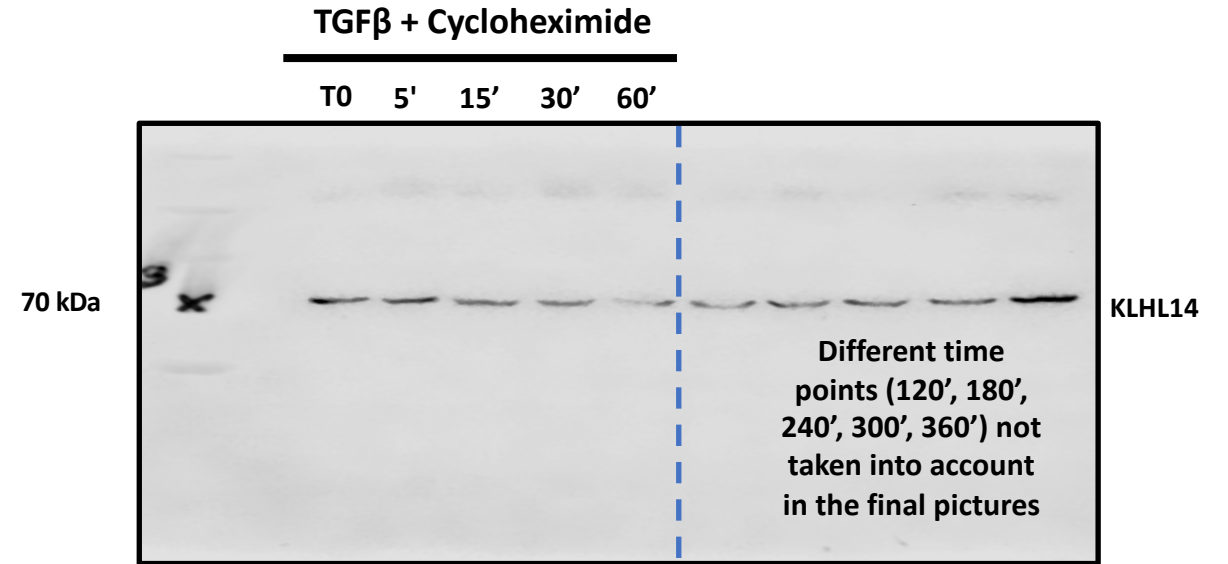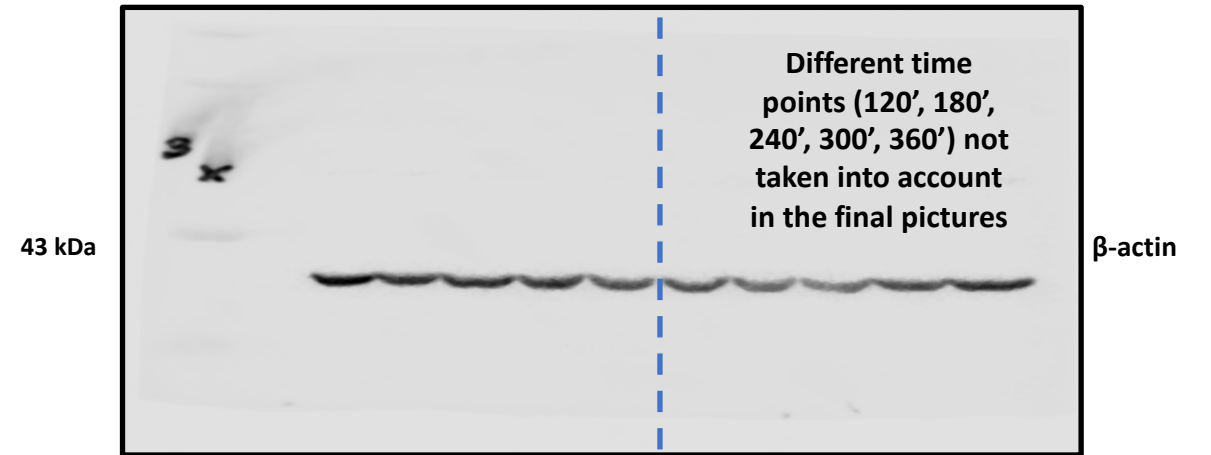

Supplement: Multimedia component 6 [file mmc6.pdf]

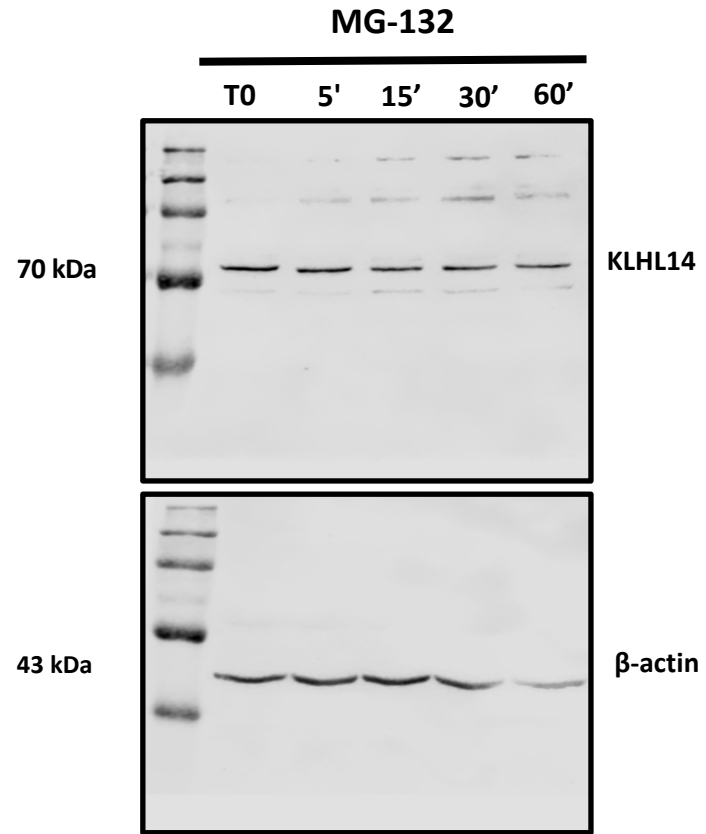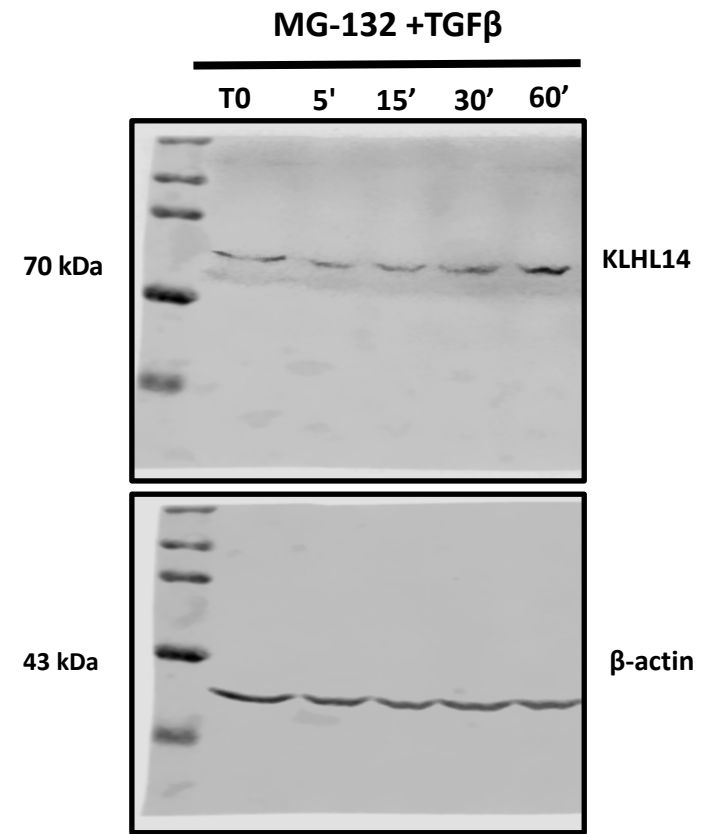

Supplement: Multimedia component 7 [file mmc7.pdf]

# Blot siRNA-KLHL14

H2052

H2452

Wound Healing

Invasion

Migration

Wound Healing

Invasion

Migration

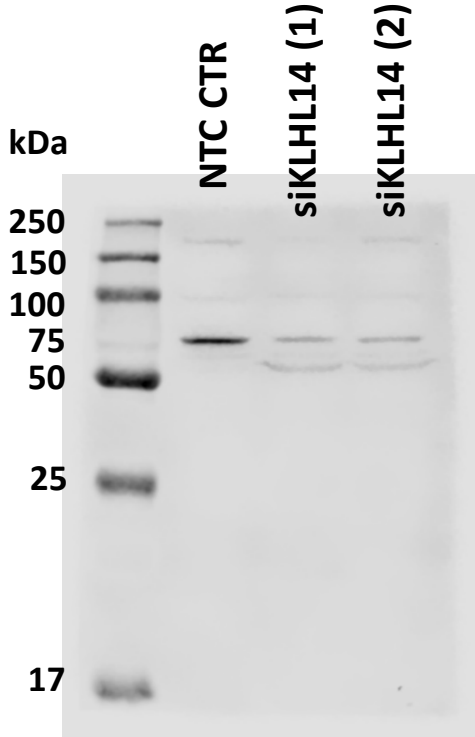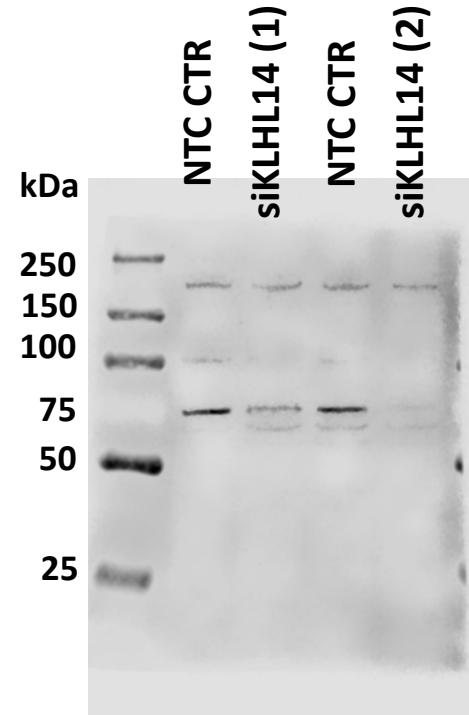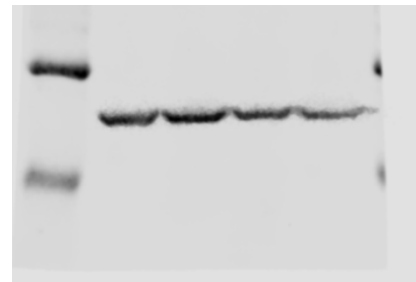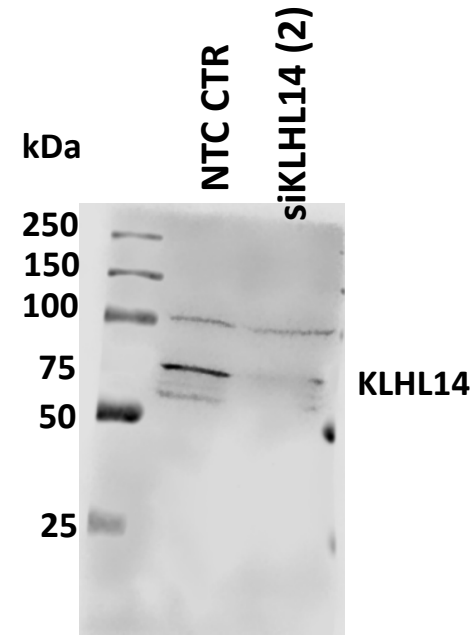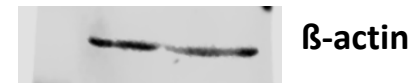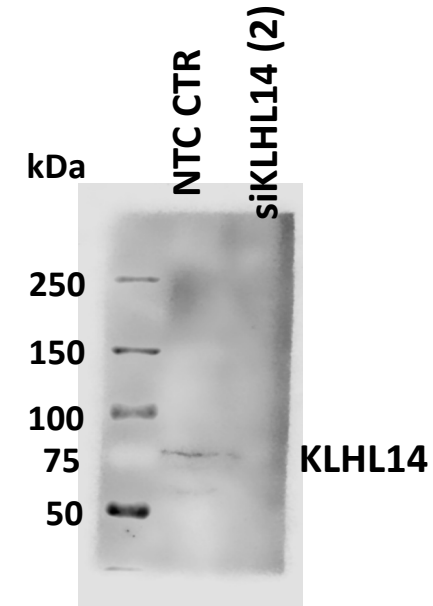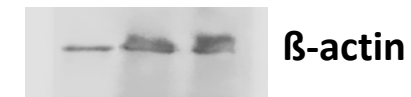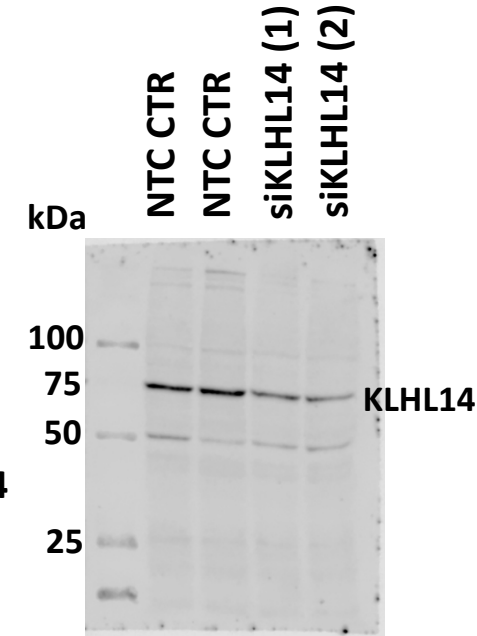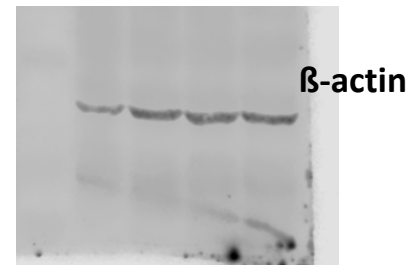

Supplement: Multimedia component 8 [file mmc8.pdf]
