# Supplementary material for: Characterization of KLHL14 anti-oncogenic action in malignant mesothelioma
Source: Heliyon. 2024 Mar 9;10(6):e27731. doi: 10.1016/j.heliyon.2024.e27731 (PMC10950656; doi:10.1016/j.heliyon.2024.e27731)
Supplement: Multimedia component 5 [file mmc5.pdf]

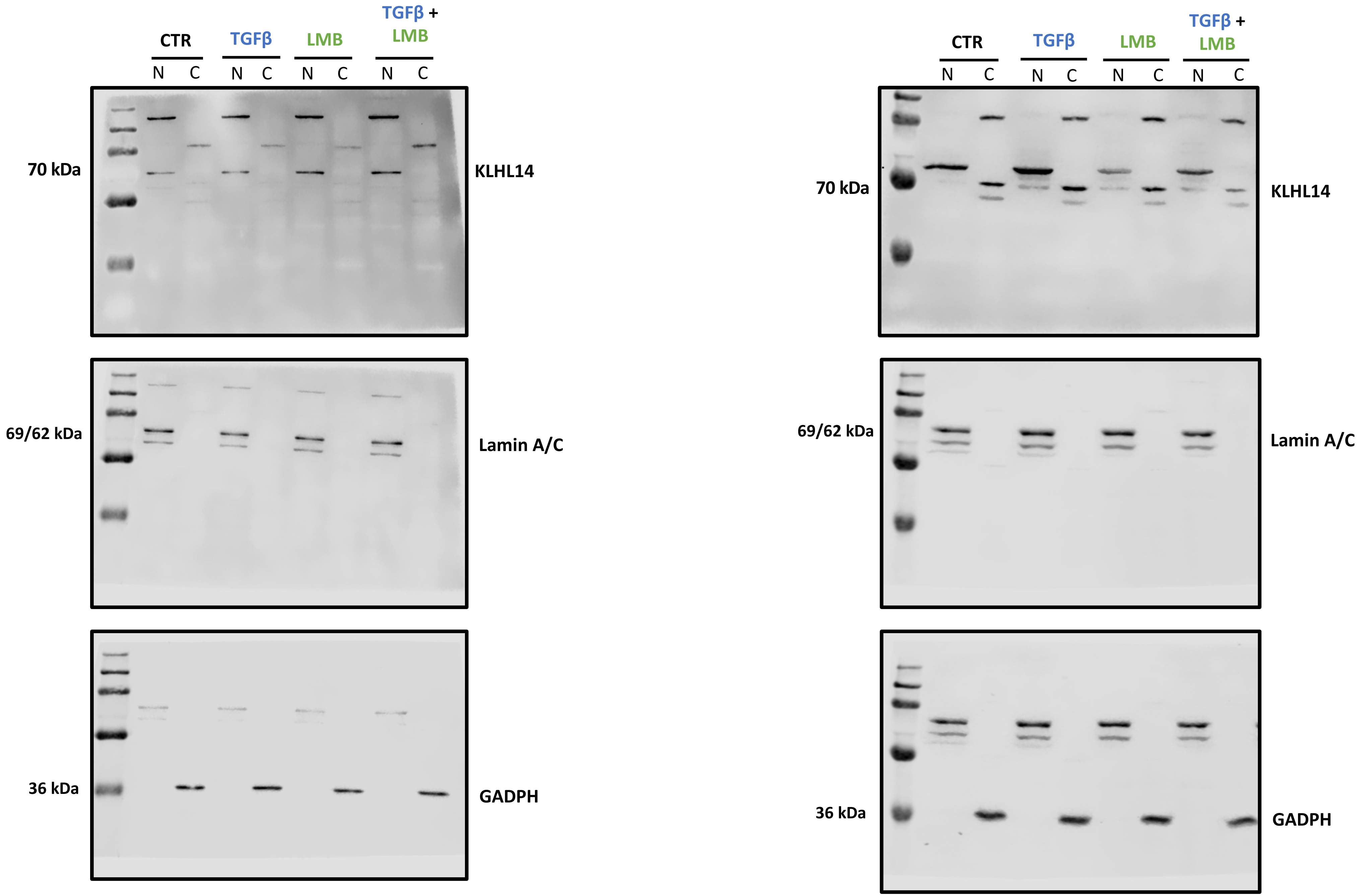
CTR
TGFβ
LMB
TGFβ + LMB
N C N C N C N C
70 kDa
KLHL14
69/62 kDa
Lamin A/C
36 kDa
GADPH
CTR
TGFβ
LMB
TGFβ + LMB
N C N C N C N C
70 kDa
KLHL14
69/62 kDa
Lamin A/C
36 kDa
GADPH
